# Expression Profile of Genes Related to Drug Metabolism in Human Brain Tumors

**DOI:** 10.1371/journal.pone.0143285

**Published:** 2015-11-18

**Authors:** Pantelis Stavrinou, Maria-Christina Mavrogiorgou, Konstantinos Polyzoidis, Vincenzo Kreft-Kerekes, Marco Timmer, Marios Marselos, Periklis Pappas

**Affiliations:** 1 Department of Neurosurgery, University of Cologne, Cologne, Germany; 2 Department of Neurosurgery, AHEPA University Hospital, Thessaloniki, Greece; 3 Laboratory of Pharmacology, University of Ioannina, Ioannina, Greece; University of Crete, GREECE

## Abstract

**Background:**

Endogenous and exogenous compounds as well as carcinogens are metabolized and detoxified by phase I and II enzymes, the activity of which could be crucial to the inactivation and hence susceptibility to carcinogenic factors. The expression of these enzymes in human brain tumor tissue has not been investigated sufficiently. We studied the association between tumor pathology and the expression profile of seven phase I and II drug metabolizing genes (*CYP1A1*, *CYP1B1*, *ALDH3A1*, *AOX1*, *GSTP1*, *GSTT1 and GSTM3*) and some of their proteins.

**Methods:**

Using qRT-PCR and western blotting analysis the gene and protein expression in a cohort of 77 tumors were investigated. The major tumor subtypes were meningioma, astrocytoma and brain metastases, -the later all adenocarcinomas from a lung primary.

**Results:**

Meningeal tumors showed higher expression levels for *AOX1*, *CYP1B1*, *GSTM3* and *GSTP1*. For AOX1, GSTM and GSTP1 this could be verified on a protein level as well. A negative correlation between the WHO degree of malignancy and the strength of expression was identified on both transcriptional and translational level for *AOX1*, *GSTM3* and *GSTP1*, although the results could have been biased by the prevalence of meningiomas and glioblastomas in the inevitably bipolar distribution of the WHO grades. A correlation between the gene expression and the protein product was observed for AOX1, GSTP1 and GSTM3 in astrocytomas.

**Conclusions:**

The various CNS tumors show different patterns of drug metabolizing gene expression. Our results suggest that the most important factor governing the expression of these enzymes is the histological subtype and to a far lesser extent the degree of malignancy itself.

## Introduction

Although modern chemotherapy has improved the prognosis of many cancers drastically, brain tumors are notorious for holding their ground. These modest results have shifted the interest towards the development of novel strategies, some attempting precise local administration of the chemotherapeutic agent to the tumor, others trying to sensitize cancer cells by transferring pro-drug activating enzymes [1,2]. In both cases, it is the tumors’ metabolic microenvironment that might play a pivotal role. It is also suggested that this environment is tissue-, cell- and patient-specific and is actually the vector of the expression of the phase I and II drug-metabolizing-enzymes (DMEs) [3].

Out of the gamut of phase I and II DMEs, we studied the expression of two of the cytochromes P450 (CYP1A1 and CYP1B1), an aldehyde dehydrogenase (ALDH3A1), the aldehyde oxidase-1 (AOX1), and three variants of glutathione-S-transferase (GSTs), namely GSTM, GSTP1 and GSTT. Although it is not completely clear to what extent the chosen genes and their corresponding proteins are implicated in the metabolism of anti-cancer drugs, and how exactly they might cluster with other related DMEs, such an exploratory approach is warranted by the trends of the recent literature.

Cytochromes P450 are the most important phase I DMEs, adding an oxygen atom to their substrate. The enzymes studied are of particular interest since they seem to have important extrahepatic function and both have been linked to various malignancies [4]. Aldehyde oxidase and dehydrogenases and their role in the metabolism of alkylating agents is currently under investigation; ALDHs seem to play a major role in mediating resistance to temozolomide, currently the primary chemotherapeutic agent for treating malignant glioma [5]. The phase II enzymes GSTs are responsible for catalyzing the formation of glutathione-S-conjugates with electrophiles inactivating and facilitating excretion of these molecules [6]. In the brain, GSTs are located primarily in astrocytes, possibly playing a neuroprotective role. On the other hand, elevated expression of GSTs may have an impact on the chemotherapeutic effect, either by direct drug metabolism or by potentially reducing the drugs ability to interact with DNA and other cellular molecules [7].

We chose to study the gene and protein expression of these seven specific DMEs to examine the metabolic footprint in various brain tumors and possible correlations between the levels of expression and types of neoplasia.

## Materials and Methods

Brain tumor specimens were obtained from a total of 77 patients undergoing primary resection without neo-adjuvant therapy. The research study was approved by the Institutional Review Board (IRB) of AHEPA University hospital and all clinical investigation was conducted according to the principles expressed in the Declaration of Helsinki. All patients consented in writing to tissue sampling and analysis. For children or patients not able to consent, written consent was obtained from the parents, caretaker or next-of-kin. All tumors had a cortical/subcortical location and no cerebellar, brain stem or spinal tumors were included. Histological interpretation of tumor type and grading was performed by an experienced neuropathologist, based on the most recent WHO classification criteria [8]. All surgical samples were snap-frozen in liquid nitrogen and stored at -80°C. Table 1 shows the basic demographic and histological data of our patient sample.

**Table 1:** Basic demographic and histological data of patient population of the present study.

Tumor Type	Number of cases	Gender (m/f)	Mean age (range)
Overall	77	41/36	58.1 (4–80)
Meningioma	30 (39%)	13/17	62 (22–79)
Meningiothelial	9	4/5	
Transitional	13	5/8	
Fibroblastic	2	1/1	
Mixed	1	f	
Psammomatous	1	f	
Lymphoplasmacytic	1	m	
Atypical	2	1/1	
Anaplastic	1	m	
Astrocytomas	27 (35,1%)	17/10	56 (4–80)
Pilocytic	3	3/0	
Diffuse fibrillary	4	4/0	
Anaplastic	1	f	
Glioblastoma	19	10/9	62.5 (42–80)
Metastasis	7 (9,1%)	2/5	57.7 (46–68)
Other Benign	7 (9,1%)	4/3	55.3 (40–79)
Schwannoma	2	0/2	
Pituitary Adenoma	1	f	
Hemangiopericytoma	2	2/0	
Hemangioblastoma	2	2/0	
Other Malignant	6 (7.7%)	5/1	52.3 (30–73)
Neuroblastoma	1	m	
Oligodendroglioma	2	2/0	
Plasmacytoma	2	2/0	
Myeloblastoma	1	m	

Total RNA and protein were isolated using the Nucleospin RNA/protein kit (Macherey-Nagel, Germany). For gene expression assays 47 samples were used, for protein levels 54 samples; samples from 32 patients were used for both assays.

### Gene Expression–qRT-PCR

RNA concentration and purity were assessed using A260nm and A260/A280 ratios respectively, at a micro-volume UV-Vis spectrophotometer (NanoDrop 2000, Thermo Scientific). *CYP1A1*, *CYP1B1*, *ALDH3A1*, *AOX1*, *GSTP1*, *GSTT1*, *GSTM3* and *VEGFA* transcript expression levels were measured in duplicates by a CFX-96-quantitative RT-PCR system (Bio-Rad Lab, Inc, CA, USA) with Taqman® Universal PCR master mix (4304437; AB, NJ, USA) using inventoried 20x assay mixes of the respective primer/probe sets (Hs01054797_g1, Hs00164383_m1, Hs00964880_m1, Hs00154079_m1, Hs00168310_m1, Hs01091674_m1, Hs00356079_m1 and Hs00900054_m1, from AB, NI, USA). For each sample, 1μg of RNA was reverse transcribed (QuantiTect® 205313; Qiagen GmbH, Hilden, Germany) in a reaction volume of 15μl for a thermal cycling program of UNG activation (50°C/2min), AmpliTaq activation (95°C/10min), denaturation (95°C/15sec) and annealing/extension (60°C/1min), for 40 cycles. Relative quantification was performed using the ΔCt method which results in ratios between our target genes and the housekeeping reference gene *β-actin* (4352935E; AB, NJ, USA).

### Protein Expression—Western Blot Analysis

SDS-polyacrylamide gel electrophoresis (PAGE) was performed in 10% separating gel with 5% stacking gel. Fifteen μl (30μg) of protein buffer from each sample was applied per lane. Gels were run at 200V for approximately 45 minutes. Subsequently, the gels were transferred by electroblotting at 400mAmp for 2 hours to a nitrocellulose membrane for immunodetection. After washing in distilled water and ponceau staining solution, non-specific binding sites were blocked by incubation of the membranes in blocking buffer (5% non-fat dry milk in TBS-Tween), at 4°C, for a minimum of 1 hour. The nitrocellulose membranes were incubated in 5% milk TBS-Tween with the -specific for the studied protein- first antibody (AOX1: 611494, DB Biosciences; CYP1A1: sc-20772, GSTM: sc-28502, GSTP1: sc-73512, GSTT: sc-32938, SantaCruz Biotechnology Inc., dilution 1:1000; and β-actin: A5441, Sigma-Aldrich, dilution 1:2000). After TBS-Tween rinsing, labeling was performed by applying the respective secondary antibody (goat anti-mouse IgG HRP: sc-2005, goat anti-rabbit IgG-HRP: sc-2004 and donkey anti-goat IgG-HRP: sc-2020, SantaCruz Biotechnology Inc.) for 2 hours, at 4°C. After rinsing again, signals were detected by enhanced chemiluminescence (ECL, Amersham, GE Healthcare, UK) by exposure to X-ray films (Fujifilm, SuperRX, Japan). Intensity of labeling was analyzed by morphometric quantification using the non-commercial Scion Imaging software.

### Statistical Analysis

To assess differences in the means of protein expression for CYP1A1, AOX1 and the three GST’s, the independent samples t-test was applied across the two main pathologies. In case any of the assumptions of parametric data was violated, the Mann-Whitney test was used. Kendall’s τ was calculated to assess how degree of malignancy correlates with the protein levels for the various DMEs.

For transcript level analysis we had three main histological categories, namely meningiomas, astrocytomas and metastases. The Kruskal-Wallis exact test was applied to check for any differences between our three categories. Mann-Whitney exact tests were used to follow up the findings and a Bonferroni correction was implemented, so all effects are reported at a 0.0167 level of significance. Again Kendall’s τ was computed to evaluate whether degree of malignancy correlates with the mRNA levels of the various DME’s.

Finally, to assess how the mRNA of five of our genes predict their protein product levels, linear regression analysis was performed.

All analysis was performed with the SPSS, version 20, running on Windows 7.

## Results

**Fig 1:**
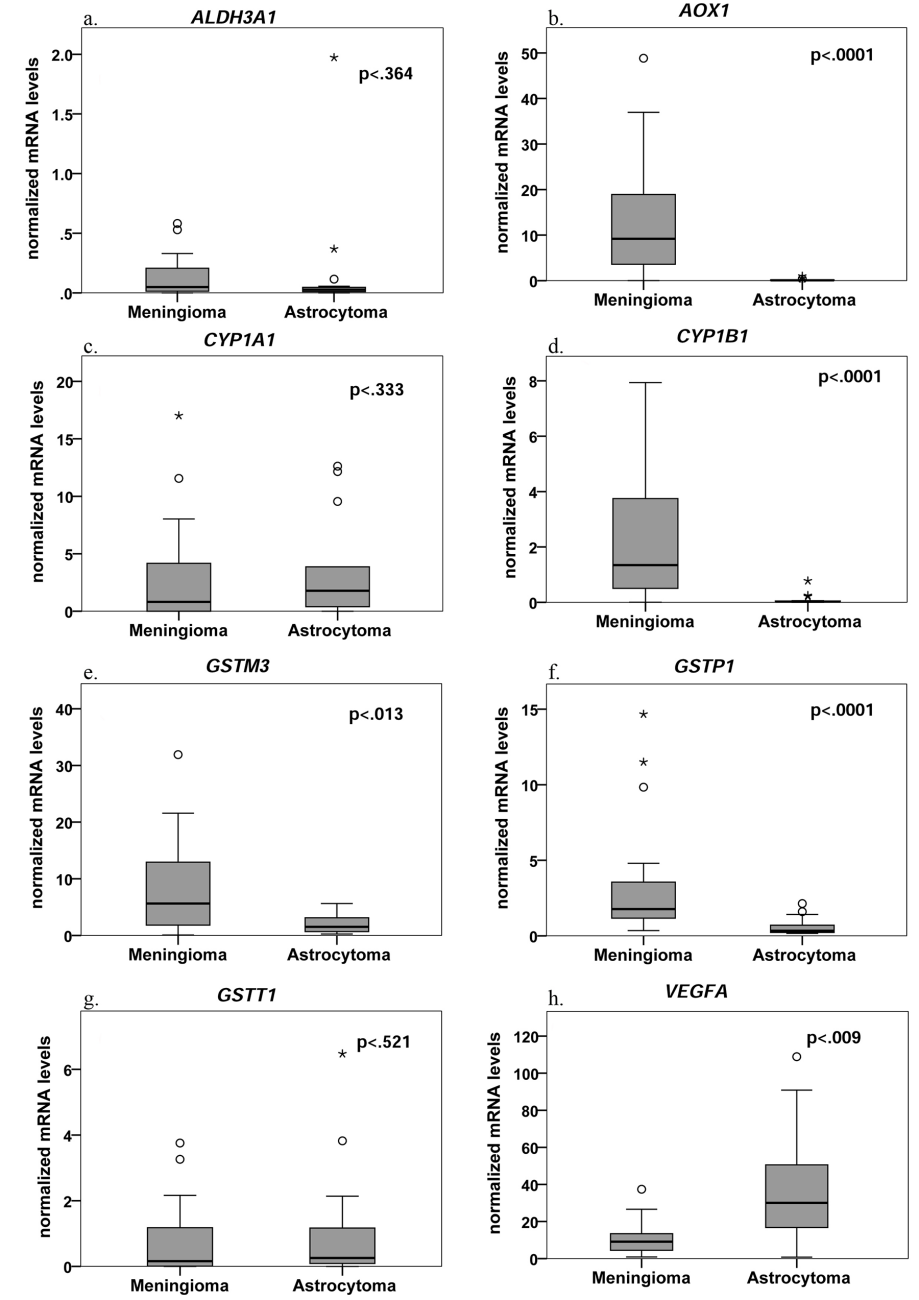
Box plots for mRNA levels of different metabolizing genes of meningiomas and astrocytomas. The line represents the median; boxes, 25% and 75% percentiles; whiskers, 1.5 times the box size. Outliers are indicated by open circle. The numbers of biologic replicates were: meningiomas, n = 20; astrocytomas, n = 15. For VEGFA the numbers of biologic replicates were: meningiomas, n = 14; astrocytomas, n = 11. The significant *p* values in each case were less than 0.05.

Using qRT-PCR, we analyzed a total of 47 tissue specimens (S1 File). Among them, there were 20 meningiomas, 15 astrocytomas and 5 brain metastases, all adenocarcinomas from a lung primary (Table 2). The mRNA levels of *AOX1*, *CYP1B1*, *GSTM3* and *GSTP1* were significantly affected by the underlying pathology (*H*_AOX1_(2) = 24.09, *H*_CYP1B1_(2) = 16.60, *H*_GSTM3_(2) = 11.96 and *H*_GSTP1_(2) = 16.52; p<0.05). No differences were found between the three tissue groups for the mRNA levels of *ALDH3A1*, *CYP1A1* and *GSTT1* (*U*_ALDH3A1_ = 3.92, p<0.14; *U*_CYP1A1_ = 1.43, p = 0.49; and *U*_GSTT1_ = 0.94, p = 0.63). Meningiomas had consistently higher mRNA levels than astrocytomas (*U*_AOX1_ = 14, p<0.001, r = -0.76; *U*_CYP1B1_ = 33.5, p<0.001, r = -0.63; *U*_GSTM3_ = 76.5, p = 0.013, r = -0.41; and *U*_GSTP1_ = 34, p<0.001, r = -0.65) (Fig 1A–1G). When comparing meningiomas with metastases, the two genes that demonstrated the highest mRNA levels, namely *AOX1* (*M* = 12.81) and *GSTM3* (*M* = 7.89) also differed statistically significantly between the two groups (*U*_AOX1_ = 5, p = 0.001, r = -0.61 and *U*_GSTM3_ = 8, p = 0.002, r = -0.57, respectively). Lastly, the comparison among astrocytomas and adenocarcinomas only revealed a significant difference for *GSTP1* (*U*_*GSTP1*_ = 8, p = 0.007, r = -0.57).

**Table 2:** Mean normalized mRNA expression of the DME genes among various tumor types^a^.

Tumor Type	n	*ALDH3A1*	*AOX1*	*CYP1A1*	*CYP1B1*	*GSTM3*	*GSTP1*	*GSTT1*
Meningioma	20	0.13 (0.04)	12.81 (2.83)	2.82 (1.02)	2.40 (0.56)	7.89 (1.84)	3.40 (0.88)	0.74 (0.25)
Astrocytoma	15	0.18 (0.13)	0.18 (0.06)	3.58 (1.19)	0.10 (0.05)	2.15 (0.48)	0.62 (0.15)	1.08 (0.47)
Metastasis	5	1.30 (1.06)	0.24 (0.09)	1.42 (0.83)	0.23 (0.16)	0.63 (0.17)	2.95 (1.34)	1.00 (0.31)
Hemangiopericytoma	2	0.06	21.88	2.77 (n = 1)	0.02 (n = 1)	3.40	3.48	1.70
Oligodendroglioma	2	0	0.05	5.21	0.06	0.59	0.59	0.02
Hemangioblastoma	1	0.09	4.08	1.95	0.09	1.05	1.26	3.88
Neuroblastoma	1	0.03	0	0.68	0.002	0.18	0.32	0.84
Schwannoma	1	0.21	0.04	0	0.004	1.04	0.34	1.61

^a^(where applicable, values in parentheses represent standard errors); n: number of samples.

In order to verify the validity of our qRT-PCR methodology, we examined the mRNA levels of VEGFA between 14 meningiomas and 11 astrocytomas. VEGFA is well known to be over-expressed in astrocytomas, but not meningiomas, and similar results would indeed serve as a "control group" for our methodology [9]. Indeed, the mean mRNA expression of astrocytomas (*M* = 38.97, *SE* = 10.43) was higher than that of meningiomas (*M* = 11.34, *SE* = 2.82) and this difference was significant (t(23) = -2.83, p = 0.009, r = 0.26). (Fig 1H).

**Fig 2:**
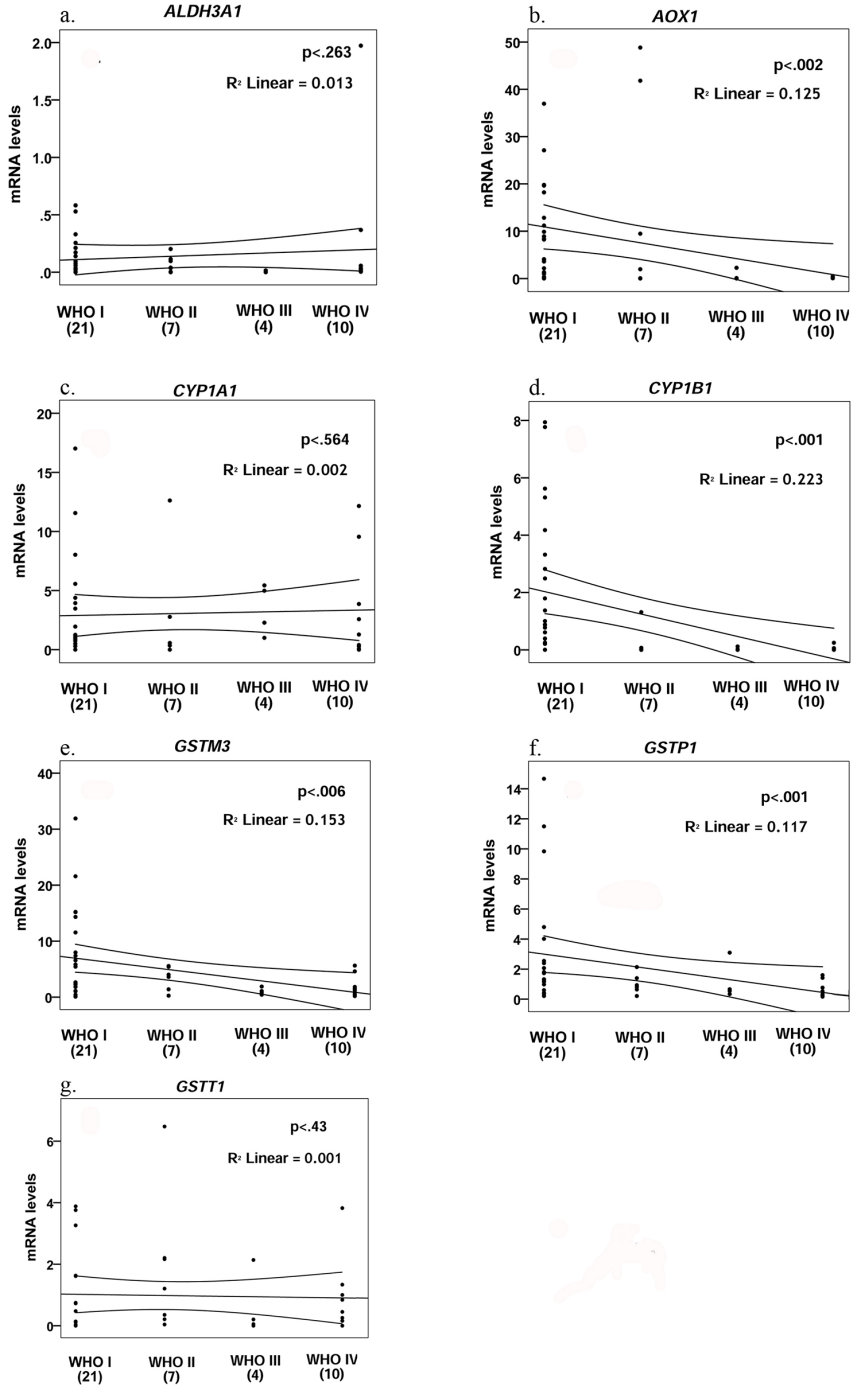
Correlation for mRNA levels and WHO tumor grade for ALDH3A1, AOX1, CYP1A1, CYP1B1, GSTM3, GSTP1 and GSTT1.

Correlation between the four WHO grades of malignancy and the mRNA expression levels was evaluated (Fig 2). Kendall’s τ test shows a significant negative correlation between malignancy grade and mRNA levels for *AOX1*, *CYP1B1*, *GSTM3* and *GSTP1* (τ = -0.37, p = 0.002; τ = -50, p<0.001; τ = -0.33, p = 0.006; and τ = -0.38, p = 0.001; respectively). This correlation was not observed for *ALDH3A1*, *CYP1A1* and *GSTT1* (τ = -0.13, p = 0.263; τ = 0.05, p = 0.564; and τ = 0.09, p = 0.43; respectively) (Fig 2).

We determined the expression of five proteins (AOX1, CYP1A1, GSTM, GSTP1 and GSTT) in 54 samples (S1 File). They were always but variably expressed in all specimens. The largest groups were those of meningiomas and astrocytomas (n = 23 and 18 respectively), with the rest of the tumor-types being represented only by one or two samples (Table 3).

**Table 3:** Mean normalized protein expression of the DME among various tumor types^a^.

Tumor Type	n	AOX1	CYP1A1	GSTM	GSTP1	GSTT
Meningioma	23	0.87 (0.05)	0.81 (0.04)	0.82 (0.05)	1.09 (0.05)	0.92 (0.09)
Astrocytoma	18	0.69 (0.05)	1.02 (0.06)	0.54 (0.05)	0.88 (0.07)	1.16 (0.10)
Metastasis	2	0.97	1.59	0.45	1.28	0.62
Hemangioblastoma	2	0.60	1.01	0.43	1.08	0.62
Plasmacytoma	2	0.45	1.11	0.26	0.65	0.68
Schwannoma	2	0.69	0.67	0.61	0.89	0.59
Adenoma	1	1.12	1.31	0.32	1.45	0.65
Hemangiopericytoma	1	0.78	1.57	0.17	0.73	0.12
Medulloblastoma	1	0.98	0.45	1.10	0.72	0.85
Oligodendroglioma	1	0.78	1.56	0.64	0.36	1.40
Neuroblastoma	1	2.40	1.26	0.23	1.57	1.02

^a^(where applicable, values in parentheses represent standard errors); n: number of samples.

**Fig 3:**
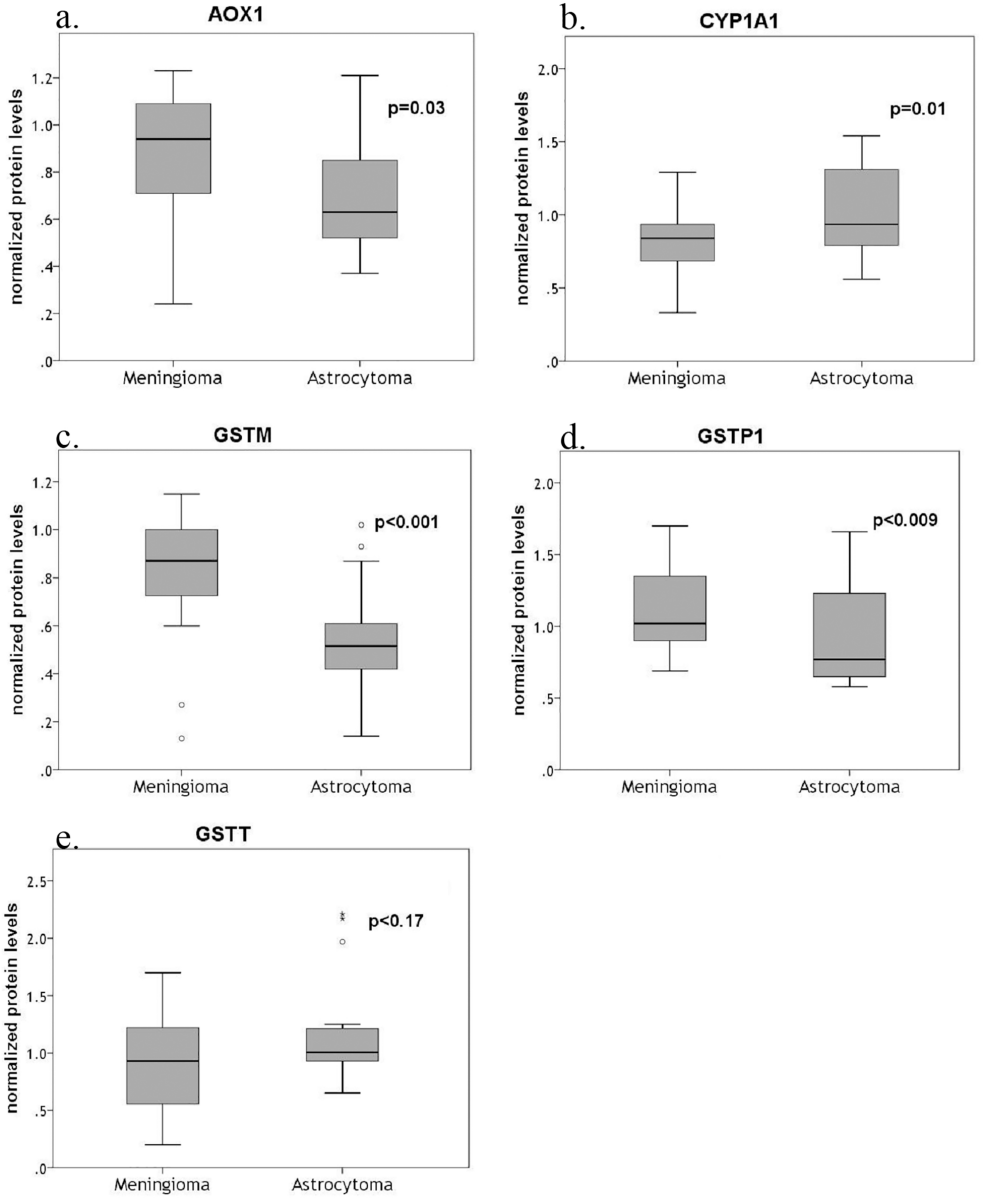
Box plots for protein levels of different metabolizing genes of meningiomas and astrocytomas. The line represents the median; boxes, 25% and 75% persentiles; whiskers, 1.5 times the box size. Outliers are indicated by open circle. The numbers of biologic replicates were: meningiomas, n = 23; astrocytomas, n = 18. The significant *p* values in each case were less than 0.05.

**Fig 4:**
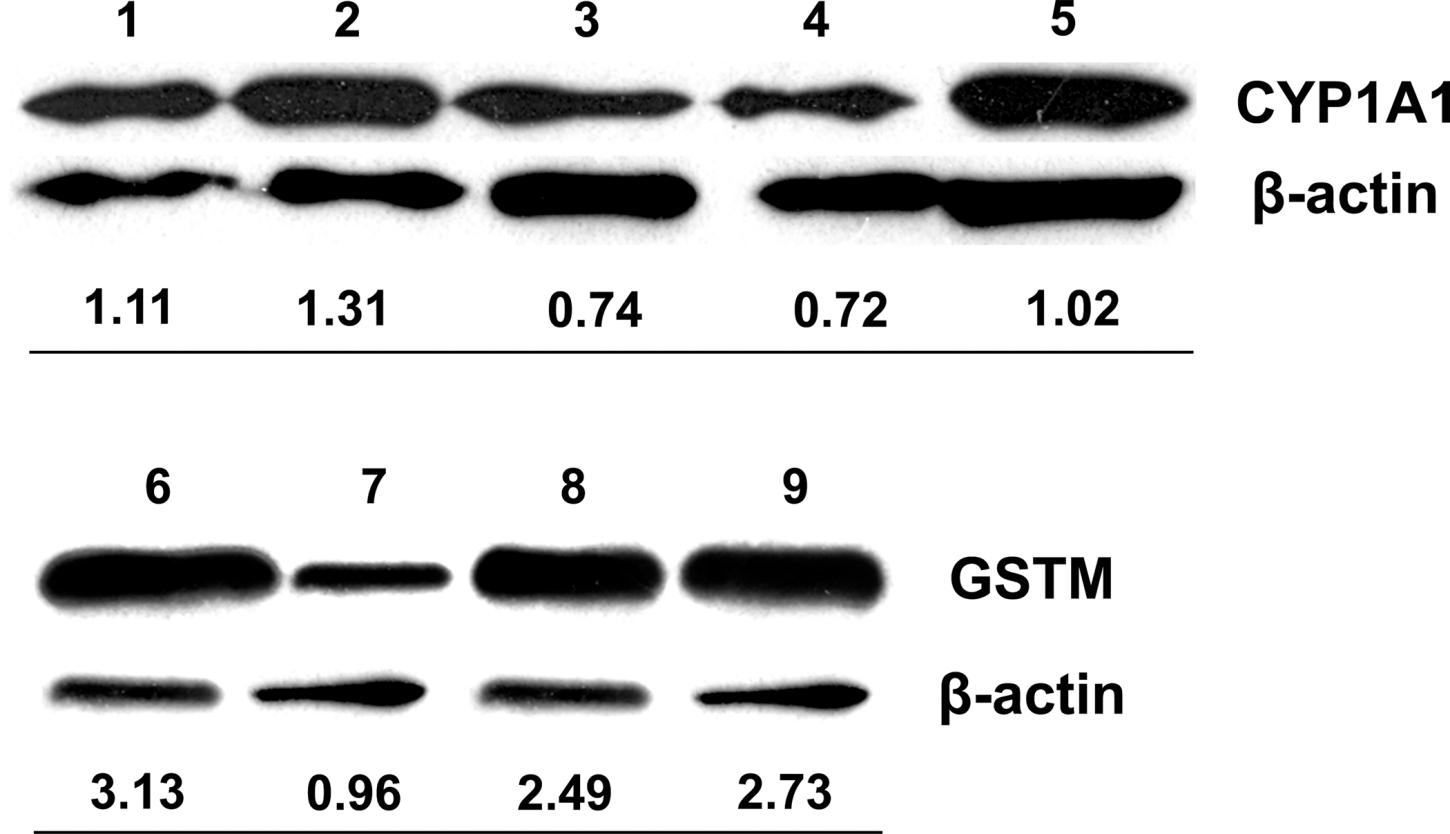
Western blotting. Normalized protein levels of CYP1A1 and GSTM for the two main tumor types (1 and 7: Glioblastoma, 2. Astrocytoma grade II, 3,4,6,8 &9: Meningioma, 5:HUVEC).

We compared meningiomas and astrocytomas. Essentially, this is a comparison between two heterogeneous cell populations, with different structure, function and lineage. There were statistically significant results between the two groups for all proteins but GSTT. On average meningiomas expressed higher AOX1, GSTM and GSTP levels than astrocytomas (AOX1: *t*(39) = 2.2, *p* = 0.03, *r* = 0.33; GSTM: *U* = 74.5, *p*<0.001, *r* = 0.54; and GSTP1: *U* = 109, *p* = 0.009, *r* = -0.4) but lower CYP1A1 levels (*t*(39) = -2.7, *p* = 0.01, *r* = 0.39) (Figs 3 and 4).

**Fig 5:**
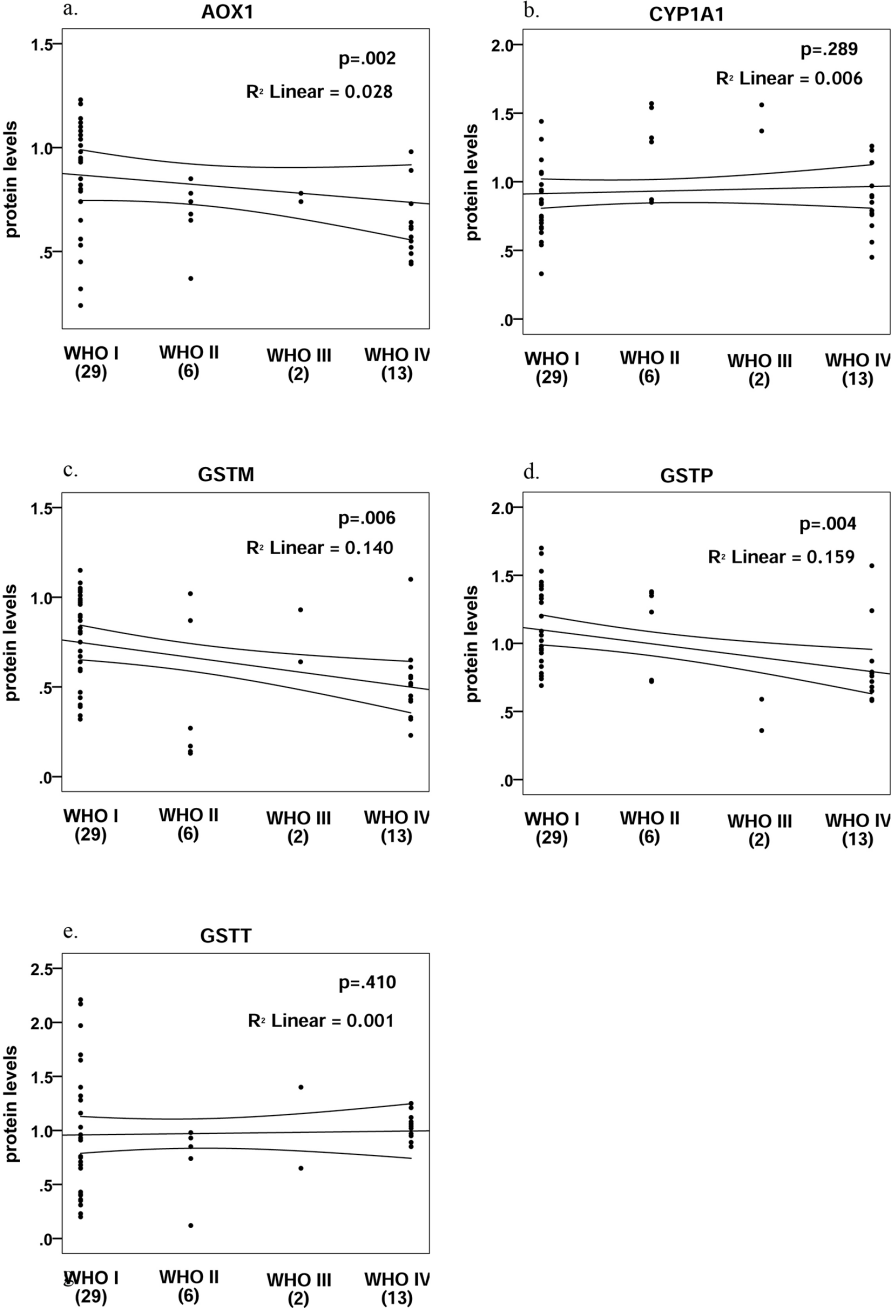
Correlation for protein levels and WHO tumor grade for AOX1, CYP1A1, GSTM, GSTP1 and GSTT.

Correlation between protein expression levels and WHO grade yielded similar results, as the ones obtained from mRNA analysis (Fig 5). Kendall’s τ test showed a statistically significant negative correlation between malignancy grade and protein levels for AOX1, GSTM and GSTP1 (τ = -0.35, p = 0.002; τ = -0.31, p = 0.006; and, τ = -0.32, p = 0.004; respectively), but not for CYP1A1 and GSTT (τ = 0.12, p = 0.289; and τ = 0.09 p = 0.410; respectively).

For 32 samples, we were able to run both qRT-PCR and western blot analysis. Linear regression assessed whether the mRNA of five of our genes can predict their protein product levels. There is no correlation for any of the studied genes for the meningiomas group, while there is positive correlation for AOX1 (F(1,10) = 10.07, p = 0.011), GSTM3 (F(1,10) = 7.9, p = 0.02) and GSTP1 (F(1,10) = 6.6, p = 0.03) in the astrocytomas group.

## Discussion

Variability in therapeutic drug responses and toxicities between tumors and patients could largely be determined by the metabolic tumor profile. DMEs have been rendered particularly important over the past years, both in cancer initiation and treatment. They have been suggested as causative factors, due to their ability to activate dietary or environmental substances to carcinogenic compounds, but they have also been researched with respect to their ability to either detoxify anticancer agents or to activate prodrugs in tumor milieu [10]. Local chemotherapy for CNS tumors is of particular interest, because of the physiologic barriers between the brain and its tumors; Carmustine impregnated wafers (Gliadel®) are already FDA-approved, while direct intracranial delivery of temozolomide by biodegradable polymers implanted locally is currently under research [11]. Maximizing the tumoricidal effect of the drug with minimal adverse effect to normal cells using customized chemotherapy, tailored to a particular tumor’s metabolic profile seems like a promising approach. This study is an effort in this direction; we examined the expression profiles of various phase I and II drug metabolizing genes and their corresponding proteins, to contribute to sketching the metabolic profile of various brain tumors.

Aldehyde dehydrogenases are phase-I enzymes, that catalyze the NAD(P)+ dependent oxidation of a wide variety of aldehydes [12]. ALDH3A1 is upregulated in various cancers and is a potential biomarker for lung cancer [13]. The 3A1 isoenzyme, is also involved in the detoxification of Cyclophosphamide (CP) and high ALDH3A1 values may be one of the reasons why CP is ineffective against gastrointestinal tumors but effective against tumors that exhibit low 3A1 expression profiles [14,15]. In our samples, the overall gene-expression of ALDH3A1 was relatively low (M = 0.26) compared to the mean expression of the other genes (data not shown) and six out of the 45 tumor samples measured, showed no expression at all. Although the metastases group had higher levels, the differences among the various tumors did not reach statistical significance. This may well be expected since the brain itself has very low basal ALDH3A1 expression, and -as discussed below- the tissue of origin might actually be the most influential factor when it comes to gene expression [16,17].

The other oxidizing enzyme for aldehydes, the AOX1, aids in the metabolism of aldophosphamide, the intermediate product of CP, to the inactive carboxyphosphamide [18]. It is also implicated in the metabolism of O6-benzylguanine (O6-BG), a substrate that inactivates O6-alkylguanine-transferase (AGT), a DNA-repair protein known to be responsible for the resistance to alkylating agents [19]. Co-administration of O6-BG with temozolomide or Gliadel® in patients with recurrent malignant glioma has shown promising results in phase II trials [20,21]. In O6-BG regimen low levels for AOX1 could indicate effective therapy and minimal resistance. In our study, meningeal tumors had higher AOX1 expression levels, compared with astrocytic tumors, both in gene and protein analysis. This difference was particularly evident when comparing the transcript product; the mean mRNA expression of meningiomas was more than 70 and 50 times higher compared with astrocytomas and metastases respectively.

Cytochrome P450s are haem-containing phase-I enzymes, mostly catalyzing oxidation reactions. The expression levels of CYP1A1 are positively linked with various malignancies, such as breast, esophageal and smoking-related lung and small intestine cancer [22–26]. Genotyping and epidemiological studies of CYP1A1 were correlated with increased risk for brain tumor [27–29]. CYP1B1 is a tumor-associated protein, which has been shown to be overexpressed in various malignant tumors [4,30]. Using immunohistochemical analysis, it was recently demonstrated that CYP1B1 is expressed in gliomas and the level of expression depends on tumor type and grade [31]. Taking advantage of the endogenous expression of CYP’s in tumors, several novel pharmacological agents are currently under development. Examples are Phortress, a synthetic CYP1A1 activated prodrug, currently undergoing phase-I trial, with potent activity against breast and colorectal cancer and resveratrol, a CYP1B1 activated cancer-preventative prodrug [32–34]. In a study by Achary, radioresistant cervical cancer cell lines were shown to overexpress the CYP1B1 gene, hinting a potential role in resistance to radiation therapy, whereas patients with various advanced-stage malignancies, vaccinated with ZYC300, a plasmid encoding an inactivated form of CYP1B1 DNA, also responded significantly better to salvation chemotherapy [35,36]. Also, the CYP1B1 gene is a target of the miR-27b which is up-regulated in gliomas samples and glioma cells compared with low grade astrocytoma cells [37,38]. In our study, meningiomas exhibited twenty-two times higher CYP1B1 mRNA expression than the average expression of all other tumors. That difference remained statistically significant when compared to astrocytomas or metastases. Although both CYP1A1 and CYP1B1 are regulated by the Ah-receptor, our data showed that CYP1A1 was strongly expressed in most of our tumor types but no significant difference was observed among the three major tumor groups [3].

A second line of defense against xenobiotic substances, are drug-glutathione conjugation reactions, by the glutathione-S-transferase enzymes, among others. Many GST gene-polymorphisms have been studied with respect to susceptibility to various brain tumors, but the results have been contradicting and inconclusive [39–44]. All three subclasses of GST tested in the present study are involved in the metabolism of carmustine. GSTP1 is the primary isoenzyme contributing to total GST activity in both normal brain and brain tumors [45,46]. Canfosfamide was developed hoping to exploit the high GSTP1 levels observed in solid tumors. It is a glutathione analog prodrug that is activated by GSTP1 and is currently undergoing phase III evaluation [47]. Comparing our two main histologic groups, we found both GSTM and GSTP1 expressed more strongly in meningiomas than in astrocytomas, both at gene and protein level. On the contrary, no such difference was observed for GSTT. We also found a negative correlation between tumor grade and mRNA as well as protein levels of GSTP. These results are in accordance with those observed recently by Wahid et al [7].

The present analysis is one of the very few that studies molecular markers at both the gene and protein level using fresh frozen tissue samples. It also explores the expression profiles of the selected enzymes in many different CNS tumors. Still, the distribution of our cases mirrors their clinical incidence, which means that various histological subgroups are inevitably underrepresented. This makes the statistical analysis within groups either impossible or dubious. In astrocytomas, when examining whether WHO-tumor-grade correlates with gene- or protein-expression levels, we found that higher tumor grades were associated with a decreased protein expression for AOX1 and CYP1A1 (data not shown). Although the level of significance was very high (P<0.01; Kendall’s τ test), the results were drawn from a total of 18 astrocytomas, out of which 11 were glioblastomas, 3 pilocytic, 3 fibrillary and only one was anaplastic, so the results have to be interpreted with caution. Similarly, when grouping all tumors into the four WHO-grades, the results were biased by the bipolar distribution of the two main tumor-types, i.e. meningiomas and glioblastomas, to the WHO I and WHO IV grades respectively. Furthermore, grouping so heterogenic tumors together under the same "WHO Grade" characterization is very liberal. The four degrees of malignancy used in clinical practice are a refined, but still arbitrary stratification rooted in the initial works of Bailey and Cushing, and which encompasses histo-pathological and clinical characteristics that may very well be not only incoherent with our genes but also with each other. Nevertheless, in absence of a more practical and clinically oriented classification we decided to adopt the aforementioned stratification. Another relevant question that should be addressed is the expression of our genes in normal brain tissue. Unfortunately, that was not possible in this study due to lack of brain tissue. Furthermore, immunohistochemical staining is needed to show whether these genes are indeed expressed in the tumor cells or the stromal elements of the tumor.

We believe that the only conclusion that can be safely drawn is that the cell of origin, i.e. a tumor’s histological lineage, is far more important than the degree of malignancy when it comes to the expression of a certain metabolic profile. This holds true especially for *AOX1*, *GSTP1* and *GSTM3* and their proteins. Finally, for all genes studied in meningiomas and for two in astrocytomas group we observed a dichotomy between mRNA levels and the translational product. In addition to transcriptional regulation, post-translational regulation through miRNAs could be responsible for the final protein expression. Such an effect has been shown, especially for CYP1B1 [37]. miRNAs are short RNA molecules that have been shown to regulate gene expression through translational repression or mRNA cleavage; and whose expression differ with the development of tumors, including glioblastomas[38].

## Conclusions

We showed that the various CNS tumors show different patterns of DME expression. Meningiomas, neoplasms studied as brain tumors, but with very different origin and behavior than astrocytomas, exhibited significantly higher expression levels for all DMEs studied. For AOX1, GSTP1 and GSTM, this was shown on both a transcriptional and translational level. Further studies are needed to determine the levels of all relevant phase I and II DMEs, the tumor-specific expression balance of which may very well govern the relative sensitivity or response to chemotherapy.

## Supporting Information

S1 File SPSS Raw-Data. (SAV)
